# Adjunctive tonic motor activation enables opioid reduction for refractory restless legs syndrome: a prospective, open-label, single-arm clinical trial

**DOI:** 10.1186/s12883-023-03462-6

**Published:** 2023-11-21

**Authors:** Mark J. Buchfuhrer, Asim Roy, Stephanye Rodriguez, Jonathan D. Charlesworth

**Affiliations:** 1 Stanford University School of Medicine, Stanford, CA USA; 2 Private Practice, Downey, CA USA; 3 Ohio Sleep Medicine Institute, Dublin, OH USA; 4 Department of Clinical Research, Noctrix Health, Inc, Pleasanton, CA USA

**Keywords:** Bioelectronics, Neurological disorder, Neuromodulation, Peripheral nerve stimulation, Opioids, Rrestless legs syndrome, Sleep disorder

## Abstract

**Background:**

There is a large population of restless legs syndrome (RLS) patients who are refractory to medication. Whereas experts recommend off-label opioids as an effective long-term treatment for refractory RLS, reducing opioid dose could substantially reduce side effects and risks. Tonic motor activation (TOMAC) is a nonpharmacological therapeutic device indicated for refractory RLS. Here, we investigated if TOMAC could enable opioid dose reduction for refractory RLS.

**Methods:**

This prospective, open-label, single-arm clinical trial [NCT04698343] enrolled 20 adults taking ≤ 60 morphine milligram equivalents (MMEs) per day for refractory RLS. Participants self-administered 30-min TOMAC sessions bilaterally over the peroneal nerve when RLS symptoms presented. During TOMAC treatment, opioid dose was reduced iteratively every 2–3 weeks until Clinician Global Impression of Improvement (CGI-I) score relative to baseline exceeded 5. Primary endpoint was percent of participants who successfully reduced opioid dose ≥ 20% with CGI-I ≤ 5. Secondary endpoints included mean successful percent opioid dose reduction with CGI-I ≤ 5.

**Results:**

On average, participants were refractory to 3.2 medications (SD 1.6) and were taking a stable dose of opioids for 5.3 years (SD 3.9). Seventy percent of participants (70%, 14 of 20) successfully reduced opioid dose ≥ 20% with CGI-I ≤ 5. Mean percent opioid dose reduction with CGI-I ≤ 5 was 29.9% (SD 23.7%, *n* = 20) from 39.0 to 26.8 MME per day. Mean CGI-I score at the reduced dose was 4.0 (SD 1.4), indicating no change to RLS severity.

**Conclusions:**

For refractory RLS, TOMAC enabled substantial opioid dose reduction without increased RLS symptoms. These results suggest that TOMAC has the potential to reduce the risk profile associated with opioid therapy for refractory RLS.

**Trial registration:**

ClinicalTrials.gov trial number NCT04698343 registered on January 6, 2021.

**Supplementary Information:**

The online version contains supplementary material available at 10.1186/s12883-023-03462-6.

## Background

Restless legs syndrome (RLS) is a neurological and sleep disorder associated with a distressing urge to move the legs that increases during immobility and worsens during the night. Nocturnal RLS symptoms result in sleep disturbances that cause sleep deprivation [1]. An estimated 2–3% of adults in the United States (US) and Europe suffer from moderate-to-severe RLS, in which symptoms present with sufficient frequency and severity to significantly reduce quality of life [2].

Refractory RLS is prevalent due to limitations of the dominant first-line medications. Dopamine agonists (DAs) provide initial relief, but long-term treatment often induces augmentation, treatment-dependent worsening of RLS symptoms associated with emergence of RLS symptoms earlier in the day [3–5]. Alpha-2-delta ligands can have limited tolerability due to side effects that include daytime sleepiness [6] and can have reduced efficacy when used to treat augmented patients [7].

Opioids are recommended for refractory RLS [6]. Results from clinical trials [8, 9] and the National RLS Opioid Registry [10, 11] demonstrate that opioids reduce symptoms of refractory RLS and that opioid doses can be maintained at a stable level with minimal dose escalation when managed by a specialist. Managed care data indicate that up to 50% of DA-treated RLS patients eventually are prescribed opioids [12]. Consensus guidelines for refractory RLS argue that benefits outweigh the dose-related risks of opioid therapy [6, 13], which include both acute side effects [14] and potential long-term risks such as opioid use disorder and overdose [15, 16].

Acute and long-term risks of opioid therapy for refractory RLS could be mitigated by reducing the opioid dose. Acute dose-related side effects are prevalent and include constipation, nausea, fatigue, and daytime sleepiness [9, 14, 17]. These side effects can limit tolerability and prevent combination therapy when other common RLS medications have an overlapping side effect profile [18]. Long-term potential risks of opioid use disorder and overdose are less frequent but more serious. Although prescribed opioid doses tend to be lower for RLS than for chronic pain, the average baseline dose from the National RLS Opioid Registry [10] exceeds the lowest risk categories established for chronic pain [15, 16, 19]. Therefore, there is opportunity to reduce risks and side effects by reducing the opioid dose in refractory RLS.

The purpose of this study was to determine the feasibility of using tonic motor activation (TOMAC) therapy to reduce the opioid dose for refractory RLS. TOMAC is a nonpharmacological therapeutic device worn bilaterally on the lower legs that electrically stimulates the peroneal nerve to engage the neuromuscular circuitry associated with voluntary leg movements such as walking or standing [20], which are known to suppress RLS symptoms. In the absence of changes to medication, TOMAC has been shown to significantly reduce RLS symptoms in refractory RLS [21, 22]. The present study represents the first evaluation of whether TOMAC treatment provides sufficient symptom relief to allow reduction of the opioid dose for patients with opioid-treated refractory RLS.

## Methods

### Study design

This study was a prospective, open-label, single-arm clinical trial conducted at two centers in the US. Active TOMAC treatment was administered throughout the study. The opioid dose was reduced iteratively until each participant completed three consecutive step-downs – lasting 2–3 weeks each – or until RLS symptoms worsened (defined below). The study was conducted in accordance with the International Conference on Harmonization guidelines on good clinical practice and the Declaration of Helsinki. An independent medical monitor was responsible for adjudicating adverse events (AEs). The study protocol and informed consent were approved by a central institutional review board (WCG Institutional Review Board). All participants provided informed consent. The trial was preregistered (ClinicalTrials.gov number NCT04698343 registered on January 6, 2021). Funding for this study was provided by NIH/NINDS R44NS117294.

### Participants

This study recruited adults aged 18–89 years with primary RLS taking prescription opioids for refractory RLS. Participants were required to maintain a stable dose of all RLS medications for the 30 days prior to study entry and a stable dose of non-opioid RLS medications throughout the study. Key inclusion criteria were baseline opioid dose ≤ 60 morphine milligram equivalents (MMEs) per day with RLS symptoms most significant in lower extremities. The MME dose limit was intended to reduce the impact of opioid withdrawal symptoms unrelated to RLS (e.g., nausea, diarrhea). Key exclusion criteria were inadequately treated primary sleep disorders other than RLS, prior experience with the study device, unstable doses of sleep medications or antidepressants, severe peripheral neuropathy affecting the lower legs, skin conditions affecting the application site, severe RLS symptoms between 10 am and 6 pm, known allergy to device materials, active medical device implants, epilepsy, dialysis, and iron-deficient anemia. All participants were from the investigators’ clinical practices except for two participants recruited from online advertisements. As prespecified in the protocol, a pilot phase consisting of 1 participant was conducted prior to the primary study and data from the pilot phase were not included in the analysis.

### Opioid dose reduction

Participants recorded daily opioid use using a written diary. For each participant, the opioid dose was reduced iteratively until the participant completed three consecutive step-downs, there was an unsuccessful step-down, or the investigator decided that additional reductions were not likely to be tolerated. A step-down was considered successful if all the following criteria were met: (1) Clinician Global Impression of Improvement (CGI-I) score ≤ 5 during final week of step-down (assessment week), (2) actual percent MME reduction during the assessment period exceeded protocol-specified thresholds (see below), (3) actual percent reduction during the assessment period was greater than the previous step-down, and (4) participant completed the step-down without participant- or investigator-initiated discontinuation.

In step-down 1, the opioid dose was reduced by ≥ 20% of the baseline dose; in step-down 2, the opioid dose was additionally reduced to ≥ 33% of the baseline dose. Step-down 3 involved a further reduction in opioid dose at the discretion of the investigator. Additionally, the minimum unit of dose reduction was ½ of the opioid pill size; for methadone, this was 2.5 mg, and thus a participant starting at 10 mg would have a step-down 1 reduction of 25% and a participant starting at 5 mg would have a step-down 1 reduction of 50%. Participants were permitted to take rescue doses of their prescribed opioid on nights when TOMAC treatment was insufficient to relieve RLS symptoms. In cases when the actual opioid dose did not match the instructed dose, the actual dose was used for efficacy endpoint evaluation and for deciding whether to continue to the next step-down. Baseline, instructed, and actual step-down opioid doses were calculated as daily MME averaged over a 1-week period. Conversion to MME followed the 2016 guidelines of the United States Centers for Disease Control and Prevention (CDC) [23].

Opioid withdrawal symptoms were assessed on a weekly basis and the investigator was permitted to discontinue the participant at any point based on clinical judgement. Each step-down included a 1–2-week run-in period followed by a 1-week assessment period during which time the opioid dose was stable. Efficacy endpoints (CGI-I and dose reduction) were evaluated based on data from assessment periods. Run-in periods provided opportunity for the investigator to adjust timing of TOMAC or opioid dosing (e.g., changing opioid dose timing from 6 to 9 pm) and for resolving any opioid withdrawal symptoms unrelated to RLS; a second run-in week was permitted if additional time was needed for either of these activities.

### TOMAC treatment

TOMAC treatment was administered throughout opioid dose reduction. Participants were instructed to self-administer 30-min sessions of TOMAC treatment whenever RLS symptoms were present. The TOMAC system (Noctrix Health, Pleasanton, CA, USA) was comprised of two therapy units worn bilaterally on the lower legs, which produced a current-controlled, charge-balanced, 4000-Hz stimulation waveform with programmable stimulation intensity of ≥ 40 milliamps. Participants were instructed to position therapy units over the peroneal nerve at the head of the fibula bone, self-administer therapy sessions whenever they experienced distressing RLS symptoms, and use a maximum of 4 sessions (120 min) per day. At study entry, trained clinic staff completed a previously described calibration procedure [20] to determine the participant-specific stimulation intensity, which was then programmed into each device for in-home use. The duration of each stimulation session was set to 30 min, after which stimulation automatically shut off.

### Outcomes and statistical analysis

Data analyses, which were primarily descriptive, were performed using Microsoft Excel.

The primary endpoint was the percentage of participants who completed step-down 1 with a CGI-I score of ≤ 5 and actual opioid dose reduction of ≥ 20%, in which both CGI-I and opioid dose reduction were assessed during step-down 1 assessment period relative to baseline (week prior to study entry). The primary endpoint and a success criterion of 50% were prespecified in the protocol prior to enrollment of the first subject. The CGI-I is a 7-point clinician-rated scale [24] that evaluates changes to RLS symptoms, in which 4 is no change, 5 is minimally worse symptoms, 6 is much worse, and 7 is very much worse. CGI-I score was selected (and prespecified) because it is designed to reliably track the clinical status of an individual participant over time. Whereas the International RLS Study Group rating scale (IRLS) [25] is the most common endpoint for the RLS status of a patient population, our prior clinical experience suggested that the CGI-I was more reliable for assessing the RLS status of an individual participant; specifically, the CGI-I was more stable than the IRLS in the absence of intervention and similarly sensitive in the presence of intervention. Post hoc analysis – described below – was conducted to evaluate the sensitivity of the primary endpoint to replacing the CGI-I success criterion with an IRLS-based success criterion. The sample size of 20 was prespecified in the research proposal for NIH/NINDS grant R44NS117294; it was selected to determine feasibility of adjunctive TOMAC treatment to reduce opioid dose for this patient population.

Maximum well-tolerated opioid reduction for each participant was defined as the largest reduction in opioid dose with a CGI-I score ≤ 5 in which both CGI-I and opioid dose reduction were assessed during assessment periods relative to baseline.

Participants who discontinued prior to step-down 1 completion for any reason were considered failures for the primary endpoint and those who discontinued prior to step-down 1 completion or had a CGI-I > 5 were counted as having 0% maximum well-tolerated opioid reduction. Other than these instances, there were no missing data. For all participants who successfully completed a step-down with a CGI-I score of ≤ 5, descriptive statistics were calculated for the CGI-I score and for change to the IRLS total score relative to baseline.

Post hoc sensitivity analysis evaluated the impact of replacing the CGI-I success criterion with an IRLS-based success criterion. For this analysis, all other statistical treatment was the same as described above for the primary endpoint.

The Pearson correlation coefficient was used to evaluate the relationships between-participant variation in maximum well-tolerated opioid reduction and the following variables: baseline opioid dose (MME), baseline IRLS total score, participant-rated RLS symptom relief during 30-min TOMAC sessions, and participant-rated RLS symptom relief during the 30-min immediately following TOMAC sessions. The latter two factors were assessed at the end of each step-down, and thus analysis of those factors excluded participants who withdrew during step-down 1 for reasons unrelated to RLS symptoms. Participant-rated RLS symptom relief was evaluated using the Patient Global Impressions of Improvement scale (PGI-I; analogous to CGI-I above) [24, 26], which was assessed at the end of step-down 1 for all participants. Exploratory assessments included asking participants to select reasons for wanting to reduce their opioid dose among multiple choice options that included reducing specific opioid-related side-effects, reducing physical dependance on opioids, concerns around risks of long-term use, concerns about availability of opioids, and tolerance.

Safety outcome measures were centrally assessed. The count and proportion of participants reporting AEs with new onset or worsening (relative to baseline) were assessed. AEs were coded and summarized at the participant level by Medical Dictionary for Regulatory Activities System Organ Class and Preferred Terms and by seriousness, severity, and relationship to the device.

Objective exposure to therapy was assessed during assessment periods through analysis of therapy unit device logs, which recorded the timing of each completed 30-min therapy session. Device logs were available for 14 of the 15 participants who successfully reduced opioid dose. Partial sessions with < 30-min duration were not included in analysis. Participants were instructed to use the device only on days with RLS symptoms and thus adherence was assessed by comparing frequency of completed sessions (from device logs) to frequency of participant reported RLS symptoms derived from an electronic daily questionnaire developed for this study (See Additional File 1).

## Results

### Participant characteristics

Participant characteristics are summarized in Table 1. On average, participants (*n* = 20) had experienced RLS symptoms for 31.1 (SD 14.1) years and were refractory to 3.2 (SD 1.6) prescription RLS medications. Most participants had failed both DAs (95%) and alpha-2-delta ligands (60%). Of the 19 participants who had failed DAs, 12 had been switched to opioid monotherapy and the other 7 were taking combination therapy. Participants had been taking opioids for RLS for an average of 8.0 (SD 4.6) years and a stable dose of opioids for an average of 5.3 (SD 3.9) years. The mean baseline IRLS score was 9.8 (SD 8.5). All participants were taking a single opioid at study entry, with 90% taking methadone and 10% taking oxycodone. The average opioid dose at study entry was 39.0 MME per day (SD 15.6), which corresponds to 9.8 mg methadone or 26 mg oxycodone.

**Table 1:** Participant characteristics

	Participants (*n* = 20)
Age, mean (SD), y	62.9 (10.2)
Sex, n (%)	
Male	12 (60.0)
Female	8 (40.0)
Race/Ethnicity, n (%)	
White/Hispanic or Latino	2 (10.0)
White/Not Hispanic or Latino	18 (90.0)
IRLS total score at baseline, mean (SD)	9.8 (8.5)
Duration of RLS symptoms, mean (SD), y	31.1 (14.1)
Number of prior RLS medications, mean (SD)	3.2 (1.6)
Prior RLS medications, n (%)	
Dopamine agonist	19 (95.0)
Alpha-2-delta ligand	12 (60.0)
Benzodiazepine	4 (20.0)
Opioid	2 (10.0)
Duration of opioid treatment for RLS, mean (SD), y	8.0 (4.6)
Baseline opioid dose, mean (SD), MME	39.0 (15.6)
Current RLS medications, n (%)	
Methadone	18 (90.0)
Oxycodone	2 (10.0)
Dopamine agonist	4 (20.0)
Alpha-2-delta ligand	3 (15.0)

*IRLS* International RLS Study Group rating scale, *MME* morphine milligram equivalent, *RLS* restless legs syndrome, *SD* standard deviation

At study entry, participants provided reasons for wanting to reduce their opioid dose. The most frequently cited reasons were to reduce physical dependence to opioids (*n* = 12 of 20, 60%), concerns about the risks of long-term use (*n* = 12 of 20, 60%), and concerns about availability of opioid medication (*n* = 14, 70%). Among the 40% of participants (*n* = 8 of 20) who wanted to reduce opioid dose due to specific opioid side-effects, the most common side effects were constipation (30%) and feelings of sedation or drowsiness (25%).

### Participant disposition

**Fig. 1:**
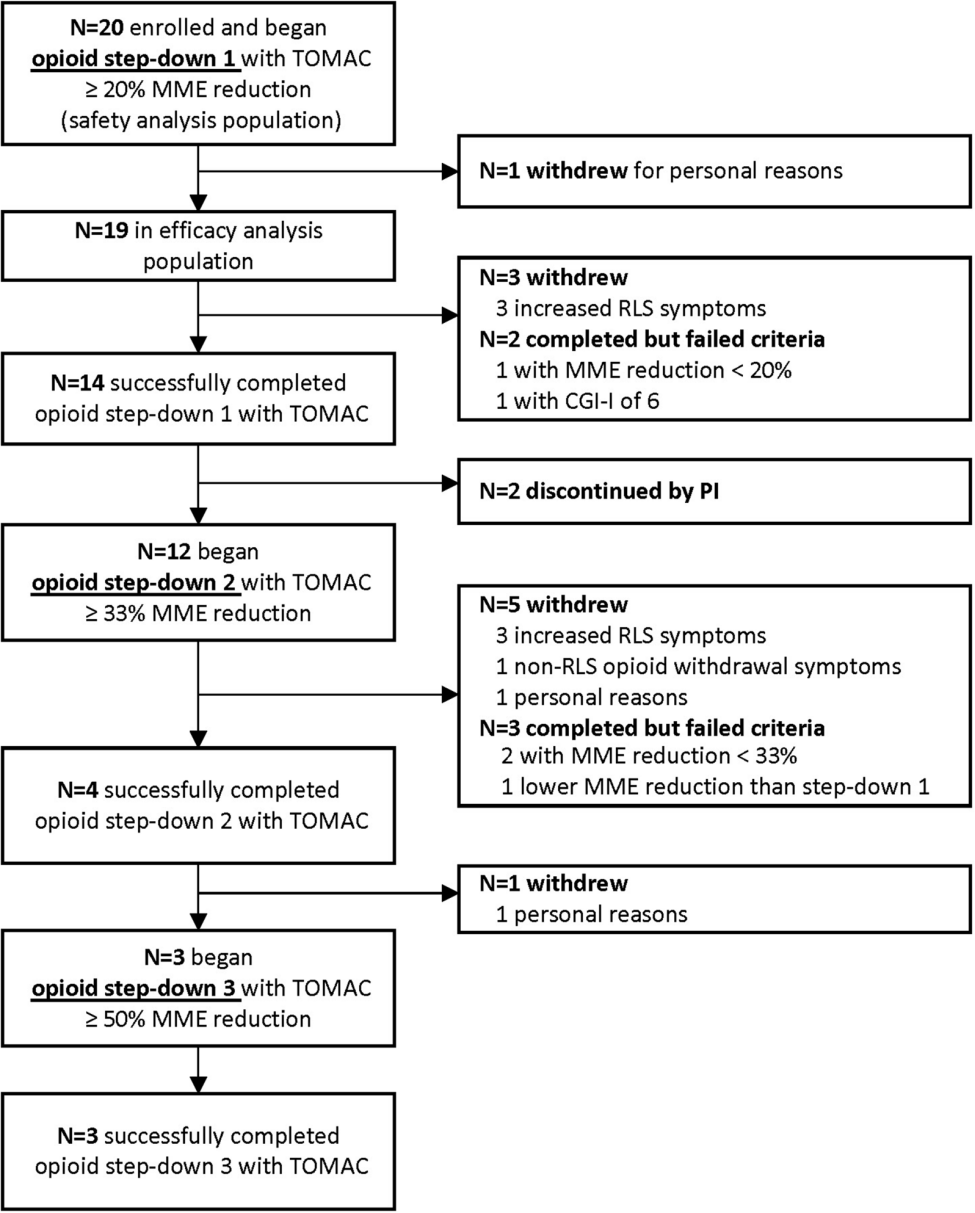
Participant disposition. During TOMAC treatment, opioid dose was reduced iteratively in three consecutive step-downs – each lasting 2–3 weeks – until RLS symptoms increased (CGI-I > 5) or the participant was discontinued for other reasons. Abbreviations: CGI-I, clinical global impression of improvement; MME, morphine milligram equivalent; TOMAC, tonic motor activation

Opioid dose was iteratively reduced in three step-downs until the participant experienced an increase in RLS symptoms that was not addressable by TOMAC treatment. All 20 enrolled participants were included in the efficacy analysis population, including three participants who withdrew due to personal reasons and one who withdrew due to opioid withdrawal symptoms unrelated to RLS. It was determined retrospectively that one participant misreported their baseline opioid dose (actual dose was 64 MME); data from this participant were included in analyses with the corrected baseline dose. Step-down completion rate (Fig. 1) was 80% for step-down 1 (16 of 20), 35% for step-down 2 (7 of 20), and 15% for step-down 3 (3 of 20), corresponding to an average study duration of 23.9 days (SD 18.3, Range 4–79).

### Primary endpoint

Of the 20 participants, 14 (70%) successfully reduced their opioid dose by ≥ 20% with a CGI-I score of ≤ 5. Thus, the percentage of participants who met the primary endpoint exceeded the prespecified success criterion of 50%. The average attempted opioid dose reduction in step-down 1 was 32.7% for the 14 successful participants and 33.3% for the 6 unsuccessful participants. Of the 6 unsuccessful participants, one completed a 17% dose reduction, one completed a 21% dose reduction with CGI-I of 6, three withdrew during step-down 1 due to increased RLS symptoms, and one withdrew for personal reasons.

### Maximum well-tolerated opioid reduction

**Fig. 2:**
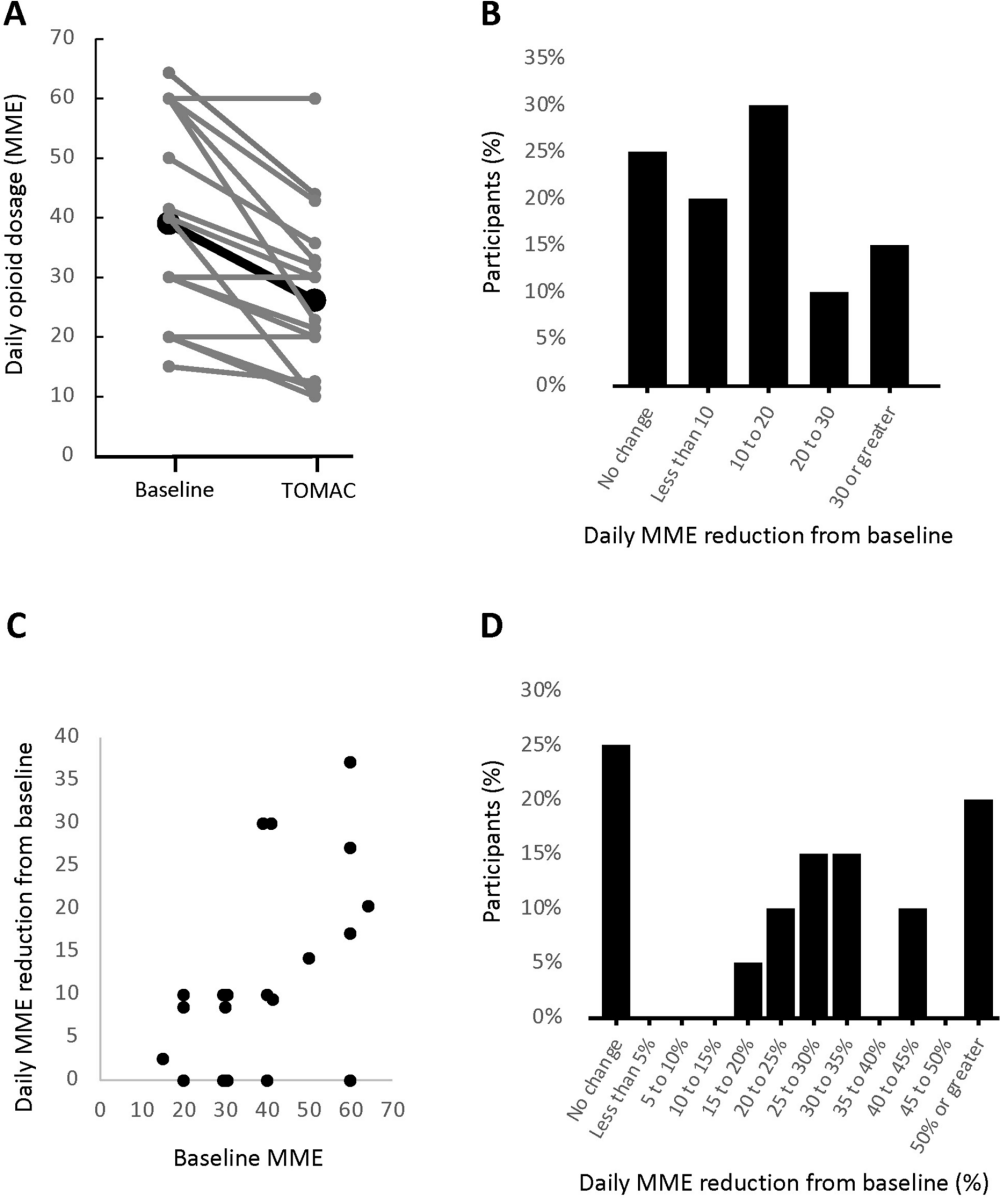
Opioid dose reduction. **A** Comparison of daily opioid dose (MME) for each participant (*n* = 20) at baseline and at the maximum successful reduction during TOMAC treatment. Each gray line represents one participant; black line represents average across all participants. **B** Histogram of maximum successful daily MME reduction from baseline. **C** Relationship between baseline MME and maximum successful daily MME reduction from baseline. Each point represents one participant. **D** Histogram of maximum successful daily MME reduction from baseline, expressed as percentage of baseline dose. In all representations, the 5 participants who did reduce opioid dose but were not able to tolerate the reduction are scored as 0 MME reduction. Abbreviations: MME, morphine milligram equivalent; TOMAC, tonic motor activation

Across the 20 participants, the average successful dose reduction was 12.3 MME (SD 11.4, Range 0–37), from 39.0 to 26.8 MME (Fig. 2A-C). Expressed as a percentage of baseline dose, the average successful opioid dose reduction was 29.9% (Fig. 2D, SD 23.7%, Range 0–75%). Baseline opioid dose was correlated with MME dose reduction (Fig. 2C, *r* = 0.53) but not percentage dose reduction (*r* = 0.16). The median duration from study entry to maximum successful opioid reduction was 21 days. For the 15 participants who had any successful opioid reduction (including the participant with a 17% reduction), the average reduction was 39.9% of the baseline dose (SD 18.3%, Range 17–75%). The maximum successful reduced dose was maintained for a period of 2–3 weeks with an actual median duration of 20 days (Mean 18.4, SD 3.3). During this time, RLS symptoms remained stable; the average CGI-I score at the reduced dose was 4.0 (SD 1.4, 95% CI 3.3 to 4.7) and the average change to IRLS score from baseline at the reduced dose was + 3.4 (SD 5.9, 95% CI 0.4 to 6.4) from a starting IRLS score of 11.9 (SD 8.5) to 15.3 (SD 6.7), remaining within the range of moderate severity RLS.

With these reductions in opioid dose, many participants crossed previously defined risk thresholds. The CDC has recommended a risk threshold of 50 MME daily opioid dose [19]; 5 of the 6 participants (83%) with a starting dose of ≥ 50 MME reduced to < 50 MME. There is also evidence for a risk threshold of 36 MME [16]; 7 of the 11 participants (64%) with a starting dose of ≥ 36 MME reduced to < 36 MME.

### Explanatory factors

**Fig. 3:**
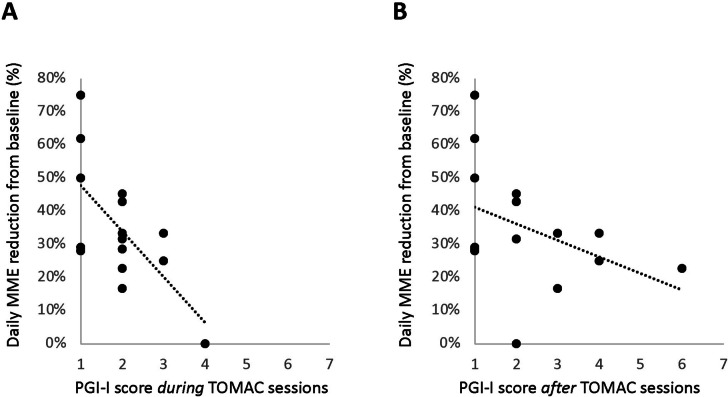
Relationship between acute TOMAC response and opioid dose reduction **A** Relationship between maximum successful daily MME reduction from baseline, expressed as percentage of baseline dose (y-axis) and PGI-I score of RLS symptom reduction during 30-min TOMAC sessions, assessed at the end of step-down 1 (x-axis). Each point represents one participant. Dashed line indicates linear regression. **B** Same as A, except PGI-I score represents RLS symptom reduction during the 30-min after the 30-min TOMAC sessions. Abbreviations: MME, morphine milligram equivalent; PGI-I, patient global impression of improvement; TOMAC, tonic motor activation

There was substantial between-participants variation in well-tolerated percentage opioid reduction during TOMAC treatment, which ranged from 0 to 75%. The most significant predictive factor of this outcome was patient-reported acute relief of RLS symptoms *during* 30-min TOMAC treatment sessions (R = -0.67, *p* = 0.006, Fig. 3A), which was measured on the PGI-I scale and assessed after each step-down, *n* = 15. This was more predictive than acute relief of RLS symptoms in the 30-min *after* TOMAC treatment sessions (R = -0.41; *p* = 0.129, *n* = 14, Fig. 3B). Baseline opioid dose (MME; R = 0.09) and baseline RLS severity (IRLS score; R = -0.15) had minimal predictive value.

### Sensitivity analysis

Next, we ran a post hoc analysis to evaluate if study endpoints were robust to incorporating participant-reported RLS symptoms in the success metric instead of clinician-evaluated RLS symptoms. In place of the clinician-evaluated CGI-I ≤ 5 success criterion, we substituted participant-rated IRLS score ≤ 20 at the end of the step-down (below the threshold for severe RLS). Based on this metric, 60% of participants (12 of 20) were successful and the average successful dose reduction was 26.4 percent. There was considerable agreement between metrics; 16 of 20 participants (80%) would have had the same outcome under both metrics (11 success, 5 fail).

### Safety

All AEs were mild (Grade 1) and non-serious (Table 2). No participants discontinued study participation due to an AE. AEs were reported by 70% of participants (*n* = 14 of 20). Device-related AEs were reported by 55% of participants (*n* = 11) and only two categories of device-related AE occurred in multiple participants: discomfort (*n* = 9, 45%) and skin irritation (*n* = 2, 10%). Discomfort refers to application-site paresthesia related to electrical stimulation and does not refer to RLS-related discomfort. During the opioid reduction procedures, multiple participants experienced increased RLS symptoms that could not be addressed by TOMAC treatment (*n* = 7, 35%) or opioid withdrawal symptoms unrelated to RLS (*n* = 2, 10%).

**Table 2:** Adverse events

	**Number (%) of Participants**	
	**All AEs**	**Device-related AEs**
Any AE	14 (70%)	11 (55%)
Serious AE	0	0
AE severity		
Grade 1	14 (70%)	11 (55%)
Grade 2 or higher	0	0
MedDRA preferred term		
Discomfort	9 (45%)	9 (45%)
Restless Legs Syndrome	7 (35%)	1 (5%)
Skin irritation	2 (10%)	2 (10%)
Drug withdrawal syndrome	2 (10%)	0
COVID-19	2 (10%)	0

MedDRA preferred terms occurring in more than 1 participant are shown

*AE* adverse event, *MedDRA* Medical Dictionary for Regulatory Activities

### Exposure to TOMAC treatment

Next, we analyzed exposure to TOMAC treatment for participants who successfully reduced opioid dose. Participants completed an average of 1.87 TOMAC sessions per day throughout the study. The frequency of TOMAC usage was similar before (1.88 per day) and during (1.83 per day) the final successful assessment week. This corresponded to an average of 18.61 completed sessions before and 7.18 sessions during the final successful assessment week.

There was high adherence with instructions to use TOMAC on days with RLS symptoms. During assessment weeks, average RLS symptom frequency was 5.9 days per week and average frequency of completed TOMAC sessions was 5.0 days per week, corresponding to an adherence ratio of 85% (*n* = 14).

## Discussion

These results indicate that TOMAC treatment can provide sufficient relief of RLS symptoms to allow opioid dose reduction in treatment of refractory RLS. The average percent opioid reduction with a CGI-I score of ≤ 5 was 29.9% (SD 23.7%) from 39.0 to 26.8 MME per day. Consistent with prior evaluations, TOMAC was safe and tolerable; there were no moderate or severe device-related AEs and no participants discontinued for reasons related to TOMAC tolerability. Participants were adherent to TOMAC treatment, using TOMAC on 85% of days with RLS symptoms. In summary, these results suggest that TOMAC is a low-risk treatment that can allow opioid dose reduction in a substantial number of RLS patients chronically treated with opioids.

The participants in this study represent some of the hardest to treat RLS patients. Most of these patients had been referred to the study investigators after they had developed severe augmentation and failed both DAs and alpha-2-delta ligands, the only two US Food and Drug Administration approved classes of RLS medication. In addition, participants had been taking a stable opioid dose for an average of 5.3 years. Data from the opioid registry indicate that reductions to opioid dose for RLS are rare in clinical practice (16% over 2 years) [11]. Therefore, the observed 70% frequency of opioid dose reduction is remarkable and unlikely to happen in the absence of TOMAC treatment.

The objective of this study was to determine feasibility of reducing opioid dosage with TOMAC as opposed to reducing RLS severity, which has been demonstrated previously [21, 22, 27]. Consistent with this objective, the eligibility criteria did not involve any limits on starting RLS severity. The baseline IRLS score (Mean 9.8, SD 8.5) indicated a study population with mild to moderate RLS. Reducing opioid dosage in this patient population with stably treated RLS has the primary benefit of reducing side-effects and risks associated with long-term opioid therapy.

The magnitude of opioid dose reduction observed in this study has the potential to reduce the side effects and risks of opioid therapy for RLS. Common dose-related side effects of opioids include constipation, nausea, fatigue, and daytime sleepiness [9, 11, 14], which are common at the starting doses observed in the study population [11]. Many participants in this study reported wanting to reduce opioid dose due to dose-related side-effects, most commonly constipation and daytime sleepiness. In addition to reducing dose-related side-effects, the dose reduction observed here could reduce the risks associated with opioid therapy. Opioid dose risk thresholds for opioid use disorder and overdose have been suggested based on data from chronic pain patients (no such data are available for RLS patients) [16, 19]. During TOMAC treatment, most participants with a baseline dose above these thresholds reduced to a dose below these thresholds, consistent with potential risk reduction. Longer-term studies with TOMAC treatment are warranted to characterize the specific benefits of opioid dose reduction for the RLS patient population.

Variation in opioid reduction among participants was associated with acute response to TOMAC treatment sessions. In clinical practice, this presents the possibility of individualizing opioid dose reduction based on acute response to TOMAC. For example, participants with a stronger acute response to TOMAC could be prescribed a more rapid reduction in opioid dose. Conversely, opioid dose reduction might be reconsidered for a patient with minimal acute response to TOMAC following proper training and calibration.

The patients in this study were representative of the opioid-treated refractory RLS population. Baseline participant characteristics were consistent with the profile of patients in the National RLS Opioid Registry [10]. The average baseline MME in this study was 39 MME compared to 38 MME in the registry. In both cases, most participants were treated with methadone, which was more common in this study (90%) than in the registry (51%). The average age was 63 years in this study and 65 years in the registry. RLS symptom severity (IRLS mean 9.8, SD 8.5) was also similar to patients in the registry (IRLS mean 13.0), suggesting that participants were on appropriate opioid doses at baseline.

Successful dose reduction in this study was defined based on a combination of investigator and participant feedback. Based on their clinical judgement, investigators evaluated changes to RLS severity on the CGI-I, determined specific dose reductions that would be attempted during each step-down, and discontinued participants if further dose reductions were unlikely to be successful. Participants had the ability to add opioid rescue doses or discontinue from the study if opioid dose reduction was not tolerable or they did not wish to continue. An alternative approach would have been to focus more heavily on participant feedback, by using the participant-rated IRLS score to evaluate RLS severity, which is a more common endpoint than the investigator-rated CGI-I in clinical trials. We conducted sensitivity analysis with an IRLS-based success metric and found similar results of a 60% success rate and 26.4% opioid dose reduction with an 80% agreement rate between metrics. This suggests that the results were robust to relying more heavily on participant feedback.

Limitations to this study include its small sample size, open-label, single-arm design, and short duration. Future work should be focused at addressing these limitations, most notably the absence of a control arm. The single-arm open-label design was selected for this study due to the risks and ethical considerations associated with opioid dose reduction in the absence of adjunctive treatment. However, this design meant that investigators and participants knew TOMAC treatment was being administered, raising the possibility of a placebo effect and bias in patient assessments. The promising results of this open-label study motivate a future randomized sham-controlled trial to directly address these limitations. A sham-controlled trial could incorporate the same sham design that was used successfully in a previous randomized controlled trial of TOMAC with stable medications [21]. Although it is possible that participants could have reduced their opioid dose in the absence of TOMAC treatment, this is unlikely because study participants were on a stable dose of opioids for multiple years prior to study entry and participants continued to have RLS symptoms (IRLS mean 9.8) at that baseline dose – suggesting that further reductions would be challenging, and reductions to opioid dose are rare in clinical practice [11]. Future work should be directed at replicating these results with more participants, more clinical centers, and a longer duration of follow-up. Due to the short duration of this study, it remains unclear how long the reduction in opioid dosage could be maintained in the event of continued TOMAC treatment. Since RLS is a chronic condition for which opioids are a long-term treatment, it would be especially important for future studies to include a maintenance or extension phase that studies the durability of reduction in opioid dosage. A recent clinical trial indicated that response to TOMAC increases with longer duration treatment [22], suggesting that TOMAC could potentially maintain long-term opioid reductions and raising the possibility that greater reductions in opioids could be possible with longer duration TOMAC treatment.

## Conclusions

Treatment of refractory RLS remains a challenge in clinical practice, primarily due to the prevalence of augmentation to DA medications [3–5]. Here, we show for the first time that TOMAC has the potential to reduce opioid use – and thus opioid-related side effects and risks – in this patient population.

### Supplementary Information

**Additional file 1.**

## Data Availability

The data that support the findings of this study are available from the corresponding author, JDC, upon reasonable request.

## Abbreviations

CDC: Centers for Disease Control and Prevention
DA: Dopamine agonist
MME: Morphine milligram equivalents
RLS: Restless legs syndrome
TOMAC: Tonic motor activation
US: United States
