# Supplementary material for: Adjunctive tonic motor activation enables opioid reduction for refractory restless legs syndrome: a prospective, open-label, single-arm clinical trial
Source: BMC Neurol. 2023 Nov 21;23:415. doi: 10.1186/s12883-023-03462-6 (PMC10662398; doi:10.1186/s12883-023-03462-6)
Supplement: Supplementary file 1 — Additional file 1. [file 12883_2023_3462_MOESM1_ESM.docx]

Participants were instructed to complete an electronic daily questionnaire with the following three questions regarding participant reported RLS symptoms:

1. Did you experience significant RLS symptoms before bedtime last night?

- Yes
- No

1. Did you experience significant RLS symptoms at bedtime last night?

- Yes
- No

1. Did you wake up in the middle of the night with significant RLS symptoms last night?

- Yes
- No

If the participant responded “Yes” to one or more of questions 1-3, then participant reported RLS symptoms were present on the previous day. If the participant responded “No” to all of questions 1-3, then participant reported RLS symptoms were not present on the previous day. If responses were missing, then the previous day was not included in analysis.
